# Supplementary material for: Osteopontin, kidney injury molecule-1, and fetuin-A as prognostic markers of end-stage renal disease: A systematic review and meta-analysis
Source: PLoS One. 2025 Apr 9;20(4):e0320804. doi: 10.1371/journal.pone.0320804 (PMC11981136; doi:10.1371/journal.pone.0320804)
Supplement: S1 File — (ZIP) [file pone.0320804.s001.zip › S1 File/4. Supplementary Material_ESRD.docx]

**SUPPLEMENTARY MATERIAL**

**S1 Table.** Search Strategy

| DATABASE | KEYWORDS | |
| --- | --- | --- |
| PubMed | #1 | “Osteopontin” OR “OPN” OR “Sialoprotein 1” OR “Secreted Phosphoprotein 1” OR “Uropontin OR |
|  | #2 | “KIM-1” OR “Kidney injury molecule-1” OR “TIMD1” OR “hepatitis A virus receptor” OR “HAV cellular receptor” OR “TIM-1” |
|  | #3 | “Fetuin-A” OR “AHSG” OR “α2-Heremans Schmid glycoprotein” |
|  | #4 | “end stage renal disease” OR “ESRD” OR “end stage kidney disease” OR “end stage kidney failure” OR “end stage renal failure” |
|  | #5 | “chronic kidney diseases” OR “chronic renal insufficiency” OR “chronic renal diseases” OR “chronic renal failure” OR “chronic kidney failure” |
|  | #6 | (#1 OR #2 OR #3) AND #4 AND #5 |
| Web of Science | #1 | Osteopontin OR OPN OR Sialoprotein 1 OR Secreted Phosphoprotein 1 OR Uropontin OR |
|  | #2 | KIM-1 OR Kidney injury molecule-1 OR TIMD1 OR hepatitis A virus receptor OR HAV cellular receptor OR TIM-1 |
|  | #3 | Fetuin-A OR AHSG OR α2-Heremans Schmid glycoprotein |
|  | #4 | end stage renal disease OR ESRD OR end stage kidney disease OR end stage kidney failure OR end stage renal failure |
|  | #5 | chronic kidney diseases OR chronic renal insufficiency OR chronic renal diseases OR chronic renal failure OR chronic kidney failure |
|  | #6 | (#1 OR #2 OR #3) AND #4 AND #5 |
| Scopus | #1 | “Osteopontin” OR “OPN” OR “Sialoprotein 1” OR “Secreted Phosphoprotein 1” OR “Uropontin OR |
|  | #2 | “KIM-1” OR “Kidney injury molecule-1” OR “TIMD1” OR “hepatitis A virus receptor” OR “HAV cellular receptor” OR “TIM-1” |
|  | #3 | “Fetuin-A” OR “AHSG” OR “α2-Heremans Schmid glycoprotein” |
|  | #4 | “end stage renal disease” OR “ESRD” OR “end stage kidney disease” OR “end stage kidney failure” OR “end stage renal failure” |
|  | #5 | “chronic kidney diseases” OR “chronic renal insufficiency” OR “chronic renal diseases” OR “chronic renal failure” OR “chronic kidney failure” |
|  | #6 | (#1 OR #2 OR #3) AND #4 AND #5 |
| Science Direct | #1 | “Osteopontin” OR “OPN” OR “KIM-1” OR “Kidney injury molecule-1” OR “TIM-1” OR “Fetuin-A” |
|  | #2 | “Cardiovascular disease” OR “Cardiovascular” |
|  | #3 | “chronic kidney diseases” |
| CINAHL | #1 | “Osteopontin” OR “OPN” OR “Sialoprotein 1” OR “Secreted Phosphoprotein 1” OR “Uropontin OR |
|  | #2 | “KIM-1” OR “Kidney injury molecule-1” OR “TIMD1” OR “hepatitis A virus receptor” OR “HAV cellular receptor” OR “TIM-1” |
|  | #3 | “Fetuin-A” OR “AHSG” OR “α2-Heremans Schmid glycoprotein” |
|  | #4 | “end stage renal disease” OR “ESRD” OR “end stage renal failure” |
|  | #5 | “chronic kidney diseases” OR “chronic renal insufficiency” OR “chronic renal diseases” OR “chronic renal failure” OR “chronic kidney failure” |
|  | #6 | (#1 OR #2 OR #3) AND #4 AND #5 |
| Proquest | #1 | “Osteopontin” OR “OPN” OR “Sialoprotein 1” OR “Secreted Phosphoprotein 1” OR “Uropontin OR |
|  | #2 | “KIM-1” OR “Kidney injury molecule-1” OR “TIMD1” OR “hepatitis A virus receptor” OR “HAV cellular receptor” OR “TIM-1” |
|  | #3 | “Fetuin-A” OR “AHSG” OR “α2-Heremans Schmid glycoprotein” |
|  | #4 | “chronic kidney diseases” OR “chronic renal insufficiency” OR “chronic renal diseases” OR “chronic renal failure” OR “chronic kidney failure” |
|  | #5 | “chronic kidney diseases” OR “chronic renal insufficiency” OR “chronic renal diseases” OR “chronic renal failure” OR “chronic kidney failure” |
|  | #6 | (#1 OR #2 OR #3) AND #4 AND #5 |
| Bioxriv | #1 | Osteopontin OR Kidney injury molecule-1 OR Fetuin-A |
|  | #2 | end stage renal disease |
|  | #3 | Chronic kidney disease |
|  | #4 | #1 AND #2 AND #3 |
| Medxriv | #1 | Osteopontin OR Kidney injury molecule-1 OR Fetuin-A |
|  | #2 | end stage renal disease |
|  | #3 | Chronic kidney disease |
|  | #4 | #1 AND #2 AND #3 |

**S2 Table**. Quality assessment of cohort studies using Newcastle Ottawa Scale (NOS) assessment.

| Author (Year) | Selection | | | | Comparability | Outcome | | | NOS Score |
| --- | --- | --- | --- | --- | --- | --- | --- | --- | --- |
|  | Representativeness of the exposed cohort | Selection of the non exposed cohort | Ascertainment of exposure | Demonstration that outcome of interest was not present at start of study | Comparability of cohorts on the basis of the design or analysis | Assessment of outcome | Was follow-up long enough for outcomes to occur | Adequacy of follow up of cohorts |  |
| Schrauben 2020 | **☆** | **☆** | **☆** | **☆** | **☆☆** | **☆** | **☆** | **-** | **8** |
| Vassallo 2019 | **☆** | **☆** | **☆** | **☆** | **☆☆** | **☆** | **☆** | **☆** | **9** |
| Sigrist 2009 | **☆** | **☆** | **☆** | **-** | **☆** | **☆** | **☆** | **☆** | **7** |
| Alderson 2017 | **☆** | **☆** | **☆** | **☆** | **☆☆** | **☆** | **-** | **☆** | **8** |
| Schmidt 2022 | **☆** | **☆** | **☆** | **☆** | **☆☆** | **☆** | **☆** | **☆** | **9** |
| Alderson 2016 | **☆** | **☆** | **☆** | **☆** | **☆☆** | **☆** | **-** | **☆** | **8** |
| Dubin 2018 | **☆** | **☆** | **☆** | **-** | **☆☆** | **☆** | **☆** | **-** | **7** |
| Hsu 2016 | **☆** | **-** | **☆** | **☆** | **☆☆** | **☆** | **☆** | **-** | **7** |
| Malhotra 2020 | **☆** | **☆** | **☆** | **☆** | **☆☆** | **☆** | **-** | **☆** | **8** |

The total maximum score is 9. A study with score from 7-9, has **high quality**, 4-6, **high risk**, and 0-3 **very high risk of bias**

**S3 Table.** Quality assessment of case-control studies using Newcastle Ottawa Scale (NOS) assessment.

| Author (Year) | Selection | | | | Comparability | Outcome | | | NOS Score |
| --- | --- | --- | --- | --- | --- | --- | --- | --- | --- |
|  | Is the case definition adequate? | Representativeness of the cases | Selection of Controls | Definition of Controls | Comparability of cases and controls on the basis of the design or analysis | Ascertainment of exposure | Same method of ascertainment for cases and controls | Non-Response |  |
| Maharem 2013 | **☆** | **☆** | **☆** | **☆** | **☆** | **☆** | **☆** | **-** | **8** |
| Kaminska 2021 | **☆** | **☆** | **-** | **☆** | **☆** | **☆** | **☆** | **-** | **6** |
| Azoz 2022 | **☆** | **☆** | **☆** | **-** | **☆** | **☆** | **☆** | **☆** | **7** |
| Kim 2013 | **☆** | **☆** | **☆** | **☆** | **☆☆** | **☆** | **☆** | **☆** | **9** |

Total maximum score is 9. A study with score from 7-9, has **high quality**, 4-6, **high risk**, and 0-3 **very high risk of bias**

**S4 Table.** Quality assessment of cross sectional studies using Newcastle Ottawa Scale (NOS) assessment.

| Author (Year) | Selection | | | | Comparability | Outcome | | NOS Score |
| --- | --- | --- | --- | --- | --- | --- | --- | --- |
|  | Representativeness of the sample | Sample size | Non-respondents: | Ascertainment of the exposure (risk factor) | Comparability of subjects in different outcome groups on the basis of design or  analysis. | Assessment of outcome | Statistical test |  |
| Caglar 2008 | **☆** | **☆** | **☆** | **☆☆** | **-** | **☆☆** | **☆** | **7** |
| Smith 2013 | **☆** | **-** | **☆** | **☆☆** | **-** | **☆☆** | **☆** | **7** |
| Mutluay 2019 | **☆** | **☆** | **☆** | **☆☆** | **-** | **☆☆** | **☆** | **8** |
| Mikami 2008 | **☆** | **-** | **-** | **☆☆** | **-** | **☆☆** | **☆** | **6** |
| Can 2021 | **☆** | **☆** | **☆** | **☆☆** | **-** | **☆☆** | **☆** | **8** |
| Zeidan 2018 | **☆** | **☆** | **☆** | **☆☆** | **-** | **☆☆** | **☆** | **8** |
| Gluba-Brzózka 2016 | **☆** | **-** | **-** | **☆☆** | **-** | **☆☆** | **☆** | **6** |
| Gluba-Brzózka 2014 | **☆** | **-** | **☆** | **☆☆** | **-** | **☆☆** | **☆** | **7** |

Total maximum score is 8. A study with score from 7-8, belong to **good studies**, 5-6 **satisfactory studies**, and 0-4 **unsatisfactory studies**

**Figure**


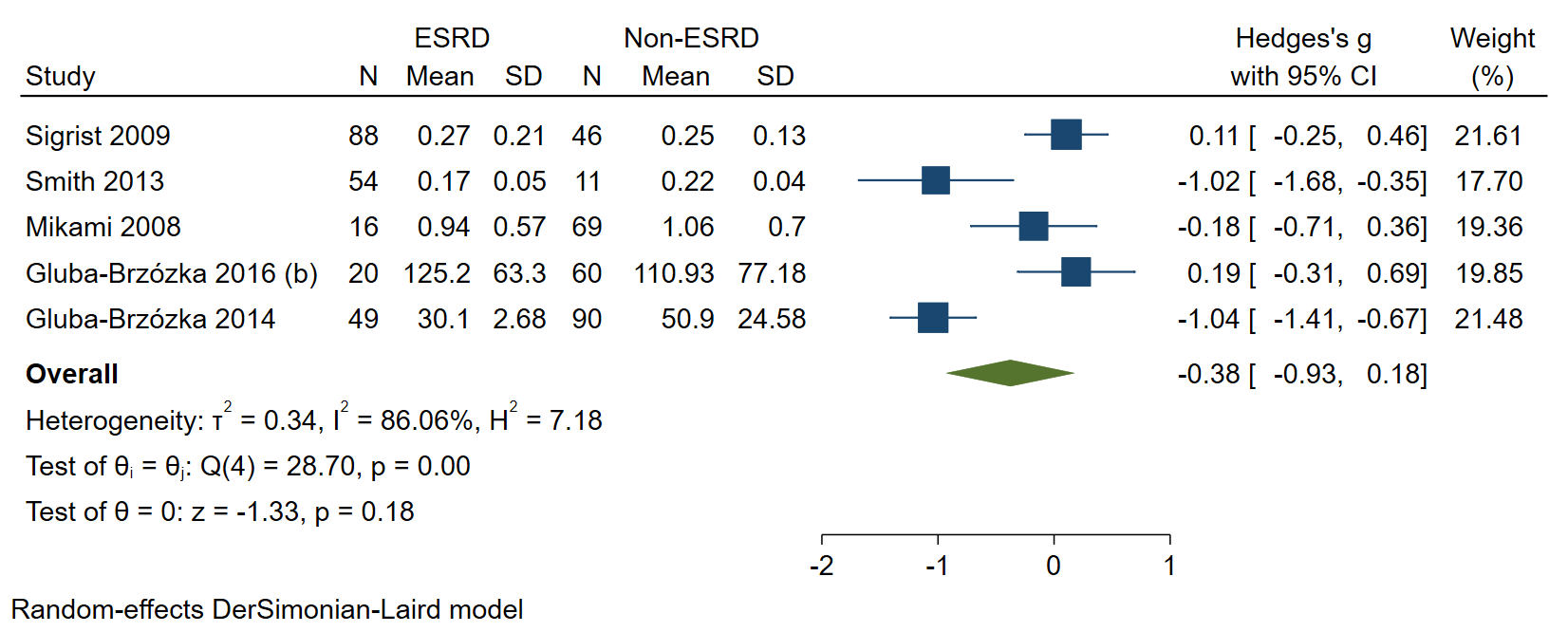


**S1 Fig.** Results of Subgroup Analysis based on Age (≥ 60 years) on the Analysis of Changes in Fetuin-A Marker Levels towards the Incidence of End-Stage Renal Disease in Chronic Kidney Failure Patients.


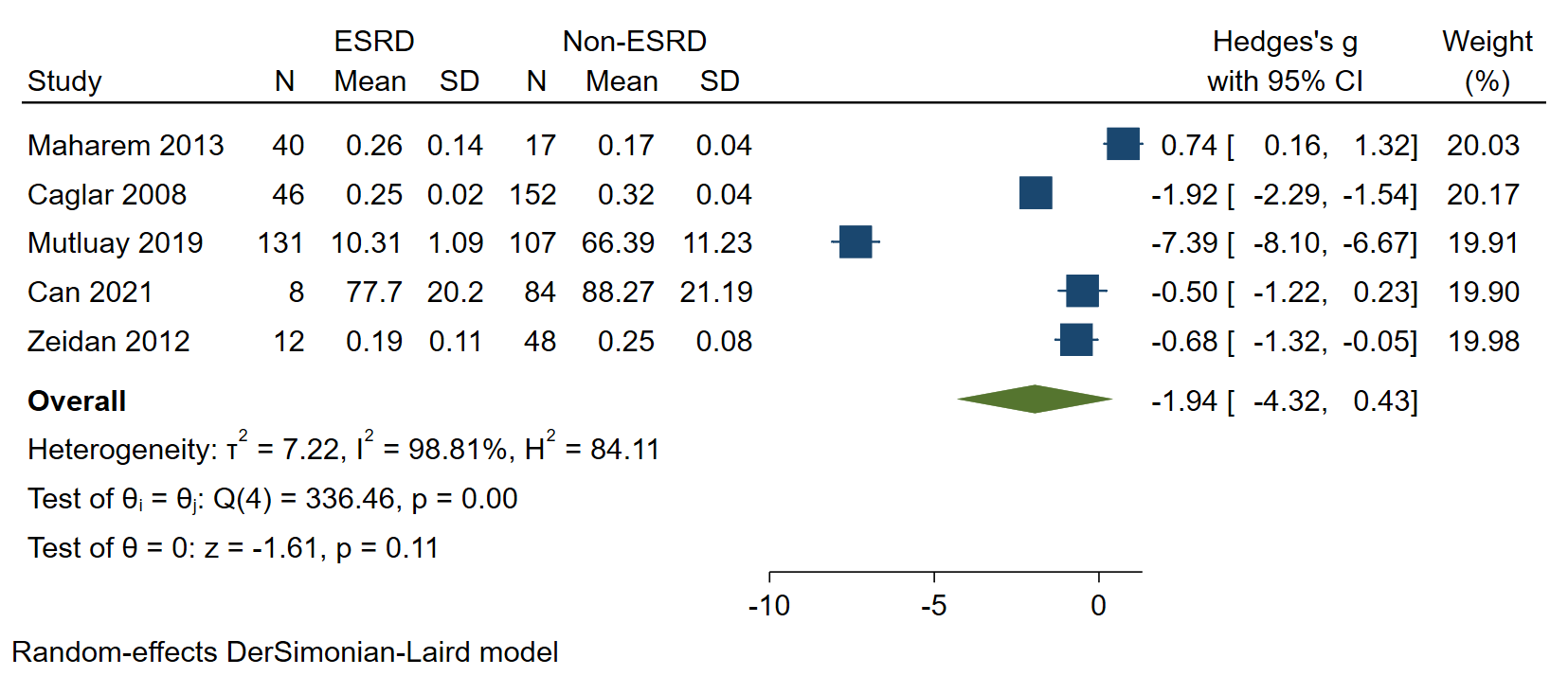


**S2 Fig.** Results of Subgroup Analysis based on Age (< 60 years) on the Analysis of Changes in Fetuin-A Marker Levels towards the Incidence of End-Stage Renal Disease in Chronic Kidney Failure Patients.


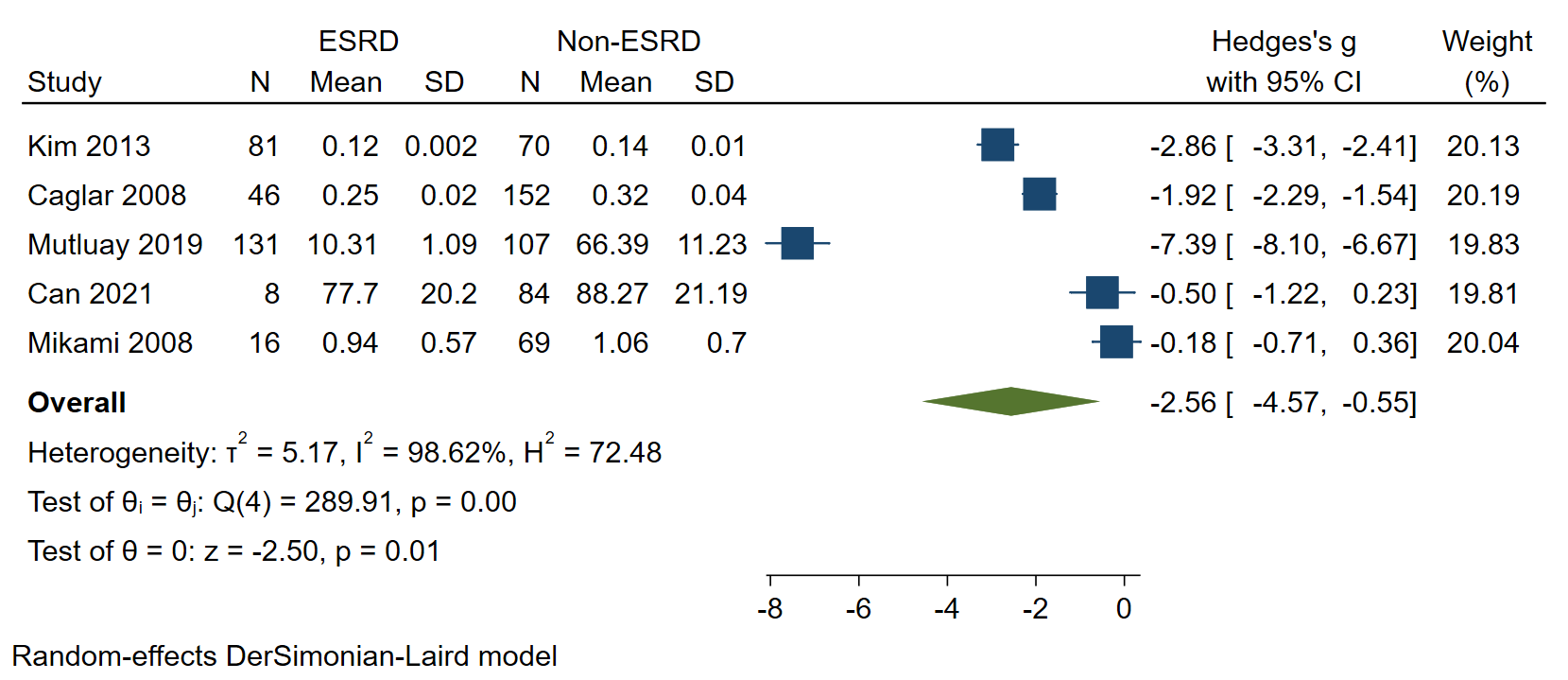


**S3 Fig.** Results of Subgroup Analysis Based on Geographical Location (Asia) in the Analysis of Changes in Fetuin-A Marker Levels towards the Incidence of End-Stage Renal Disease in Chronic Kidney Failure Patients.


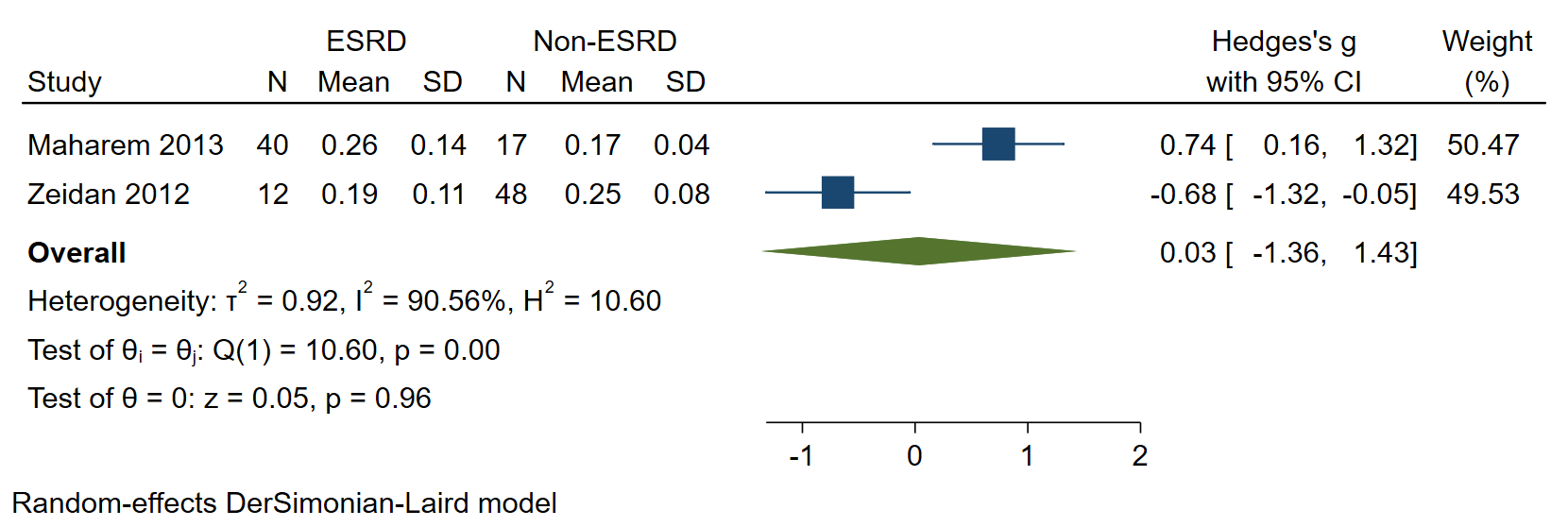


**S4 Fig.** Results of Subgroup Analysis Based on Geographical Location (Africa) in the Analysis of Changes in Fetuin-A Marker Levels towards the Incidence of End-Stage Renal Disease in Chronic Kidney Failure Patients.


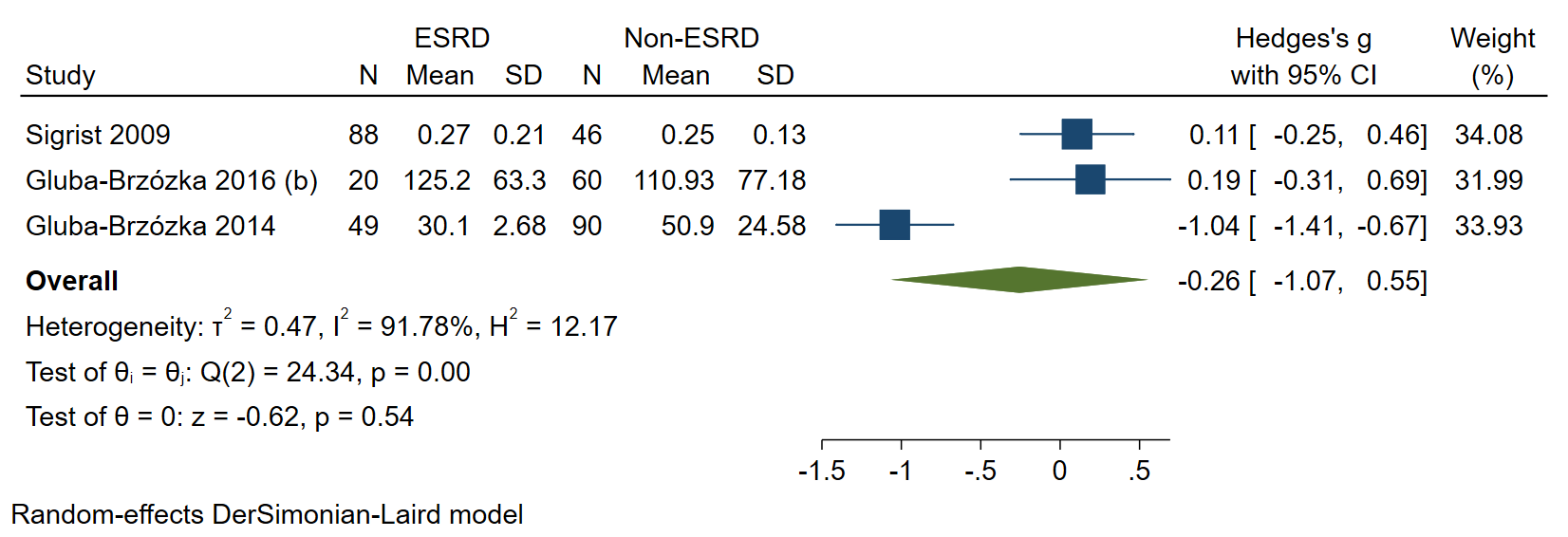


**S5 Fig.** Results of Subgroup Analysis Based on Geographical Location (Europe) in the Analysis of Changes in Fetuin-A Marker Levels towards the Incidence of End-Stage Renal Disease in Chronic Kidney Failure Patients.


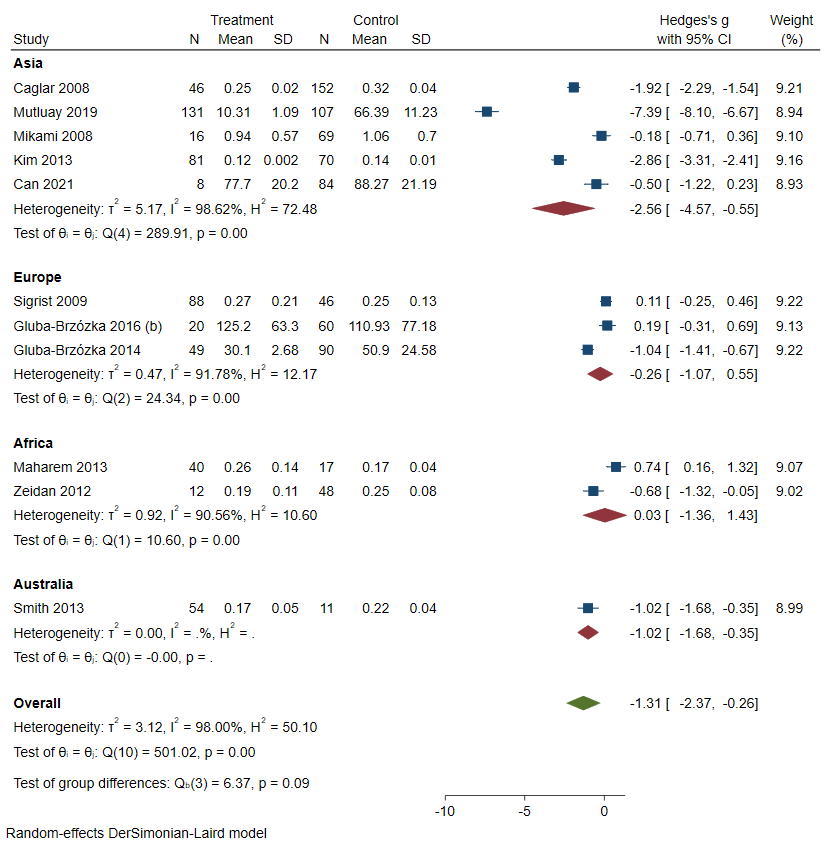


**S6 Fig**. Results of Subgroup Analysis Based on Geographical Location (Overall) in the Analysis of Changes in Fetuin-A Marker Levels towards the Incidence of End-Stage Renal Disease in Chronic Kidney Failure Patients.


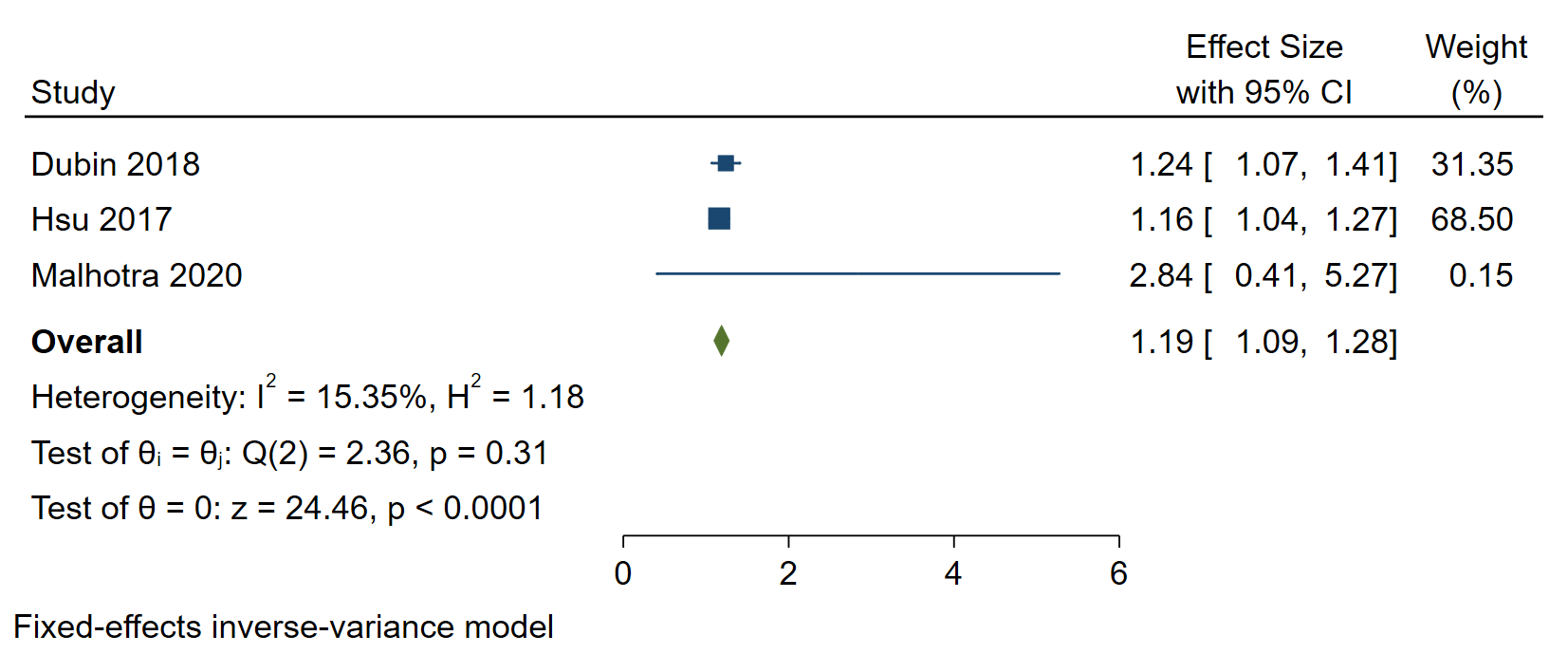


**S7 Fig.** Subgroup Analysis Result based on Specimen Type (Urine KIM-1) on the Analysis of Changes in KIM-1 Marker Levels towards the Incidence of ESRD in Chronic Kidney Disease Patients


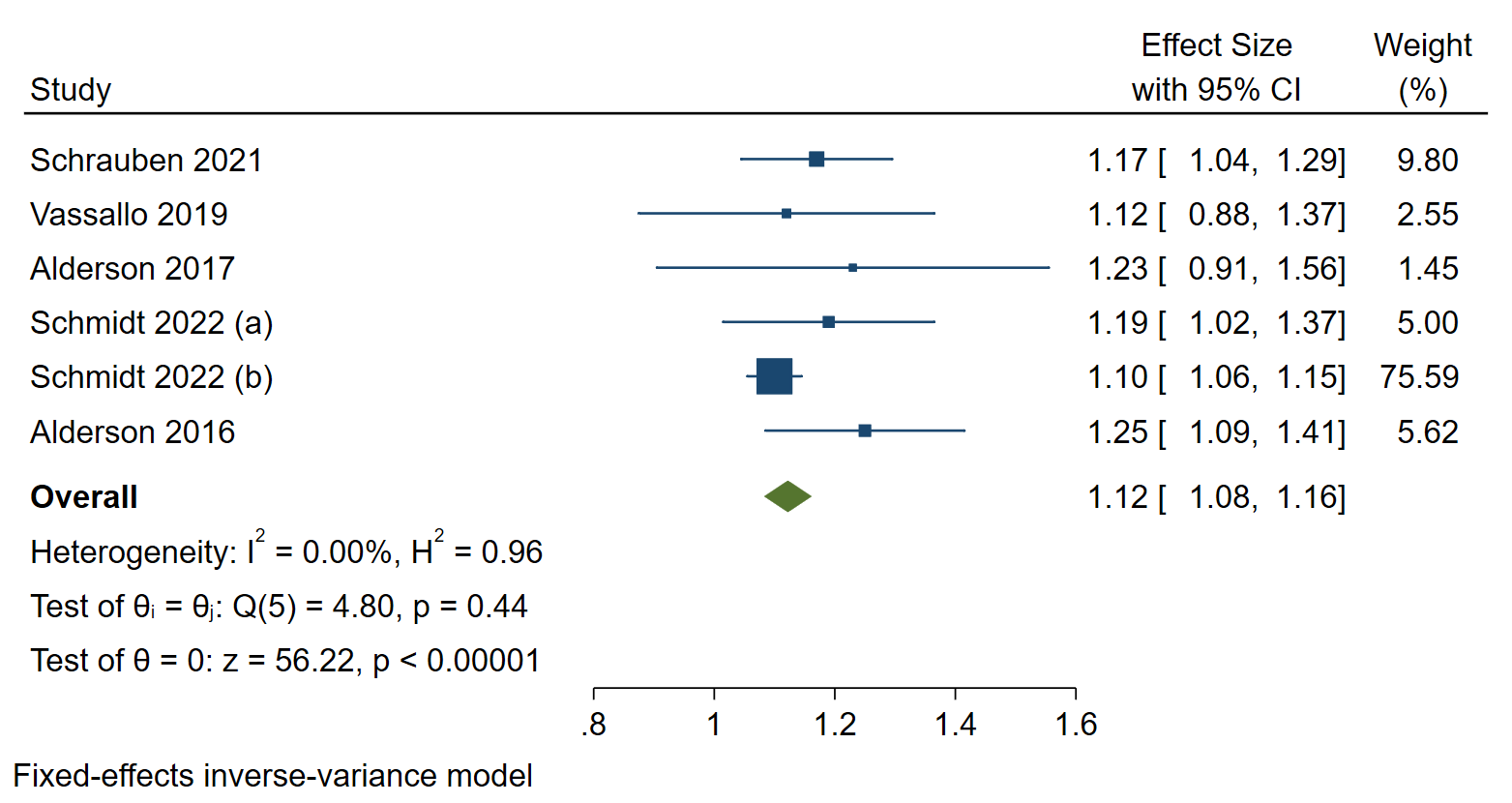


**S8 Fig.** Subgroup Analysis Result based on Specimen Type (Blood KIM-1) on the Analysis of Changes in KIM-1 Marker Levels towards the Incidence of ESRD in Chronic Kidney Disease Patients
